# Case Report: Revisiting the Internal Mammary Artery Perforator Flap: Salvage Option for Circumferential Pharyngo-Esophageal Defects

**DOI:** 10.3389/fsurg.2021.638345

**Published:** 2021-03-17

**Authors:** Nicholas Marsden, Lipi Shukla, Damien Grinsell

**Affiliations:** ^1^Department of Plastic Surgery, Royal Melbourne Hospital, Parkville, VIC, Australia; ^2^Department of Plastic Surgery, St. Vincent's Hospital, Fitzroy, VIC, Australia

**Keywords:** IMAP flap, pharyngo-esophageal reconstruction, stricture, fistula, radiotherapy

## Abstract

Patients that present with pharyngeal strictures and pharyngocutaneous fistulas in the context of previous reconstruction and post-operative radiotherapy often report significant morbidity and reduction in quality of life. Reconstruction of such defects present a substantial clinical challenge requiring the importation of unirradiated vascularized tissue to facilitate healing in a friable, fibrotic, and vessel depleted tissue bed. The authors present a case report demonstrating an adaptation of the internal mammary artery perforator (IMAP) flap for reliable reconstruction of circumferential pharyngeal defects with primary tension free closure of the donor site. This technique avoids the use of free tissue transfer in a hostile, irradiated neck. The tubed IMAP flap is an excellent option, serving the purposes of reconstruction as well as addressing the patient's presenting issues of a chronic sinus and pharyngeal stricture inhibiting oral intake.

## Introduction

Stricture at the cutaneous-mucosal anastomotic junction between the pharynx and tubed skin flaps can be a difficult problem to manage following pharyngo-esophageal reconstruction (1). This reconstructive dilemma is further complicated in the setting of previous radiation and neck dissection, often leaving hostile surgical conditions and vessel deplete necks. Shayan et al. previously described the pedicled internal mammary artery perforator (IMAP) fasciocutaneous flap as a useful technique for reconstructing small-medium partial defects following annular pharyngeal stricture release (2). We describe a case of a circumferential pharyngo-esophageal defect following releasing of a pharyngeal stricture, in a patient who had previously had an antero-lateral thigh (ALT) reconstruction, bilateral neck dissections, and radiotherapy. This is the first case reporting the successful use of the tubed pedicled IMAP flap to reconstruct a circumferential defect.

## Case Description

### Patient Information

A 71 year-old male presented with a pharyngocutaneous fistula on the background of a stage 4 (T4aN2M0) right piriform fossa Squamous Cell Carcinoma (SCC) managed with laryngectomy, partial pharyngectomy, bilateral neck dissections, and reconstruction of a neopharynx using an ALT flap. The patient received 56Gy post-operative radiotherapy and adjuvant chemotherapy.

### Clinical Findings

The patient presented a chronic non-healing wound (a common and difficult problem in irradiated tissue) and progressive reduction in oral intake. Having undergone serial balloon dilatation for management of his neo-pharyngo-esophageal stricture and concurrently developing a pharyngocutaneous fistula 3 years post operatively, the patient underwent intraoperative stricture release and excision of fistula, leaving a 4 cm circumferential defect.

### Operative Technique

**Figure 1:**
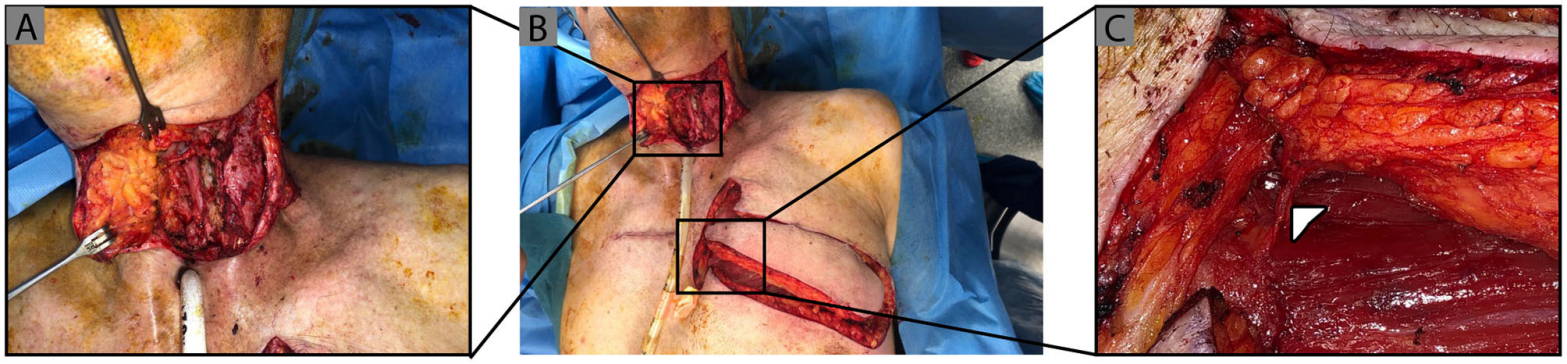
Pharayngocutaneous fistula defect and IMAP flap raised on left 2nd intercostal perforator. Clinical photographs demonstrating **(A)** circumferential full thickness pharyngeal defect post scar and stricture release, followed by **(B)** left sided IMAP flap raised and islanded on a second intercostal perforator (Δ) which is shown with further magnification in **(C)**.

**Figure 2:**
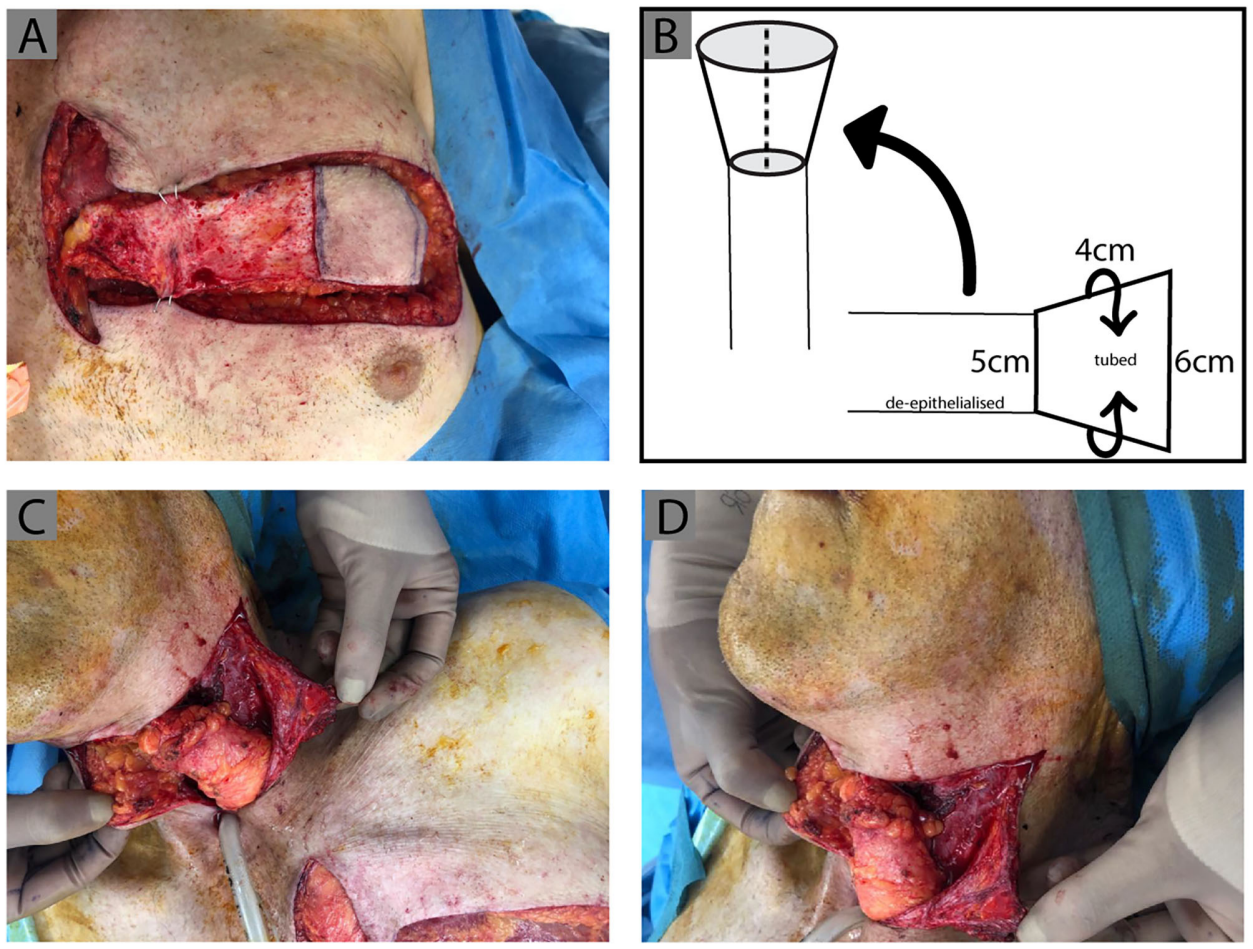
IMAP tube design. Clinical photographs demonstrating the process of IMAP tubing, de-epithelialization and inset. **(A)** The medial extension of the flap is de-epithelialized and the lateral portion is tubed with the epithelial surface forming the internal tube lining. This is diagrammatically represented with the measurements of the flap design used in this case in **(B)**. **(C,D)** The flap was then rotated superiorly and a wide subcutaneous tunnel created, insetting the tubed IMAP proximally to the pharynx (previous tubed ALT flap) and distally to the esophagus.

Given the hostile nature of his irradiated, previously dissected and fibrotic neck tissue, an IMAP flap was preferred rather than free tissue transfer for reconstruction. Intraoperative exploration demonstrated a suitable IMA/V perforator in the left 2nd intercostal space, which was utilized to raise an IMAP flap (Figure 1). The flap was extended to the midaxillary line, raised in a suprafascial plane, with paddle dimensions of superior width 6 cm, inferior width 5 cm, and a length of 4 cm and tubed on the chest. After de-epithelialization and islanding, the flap was tunneled across a short skin bridge, inset into the defect over a nasogastric tube and the external defect was closed (Figure 2).

### Outcomes

**Figure 3:**
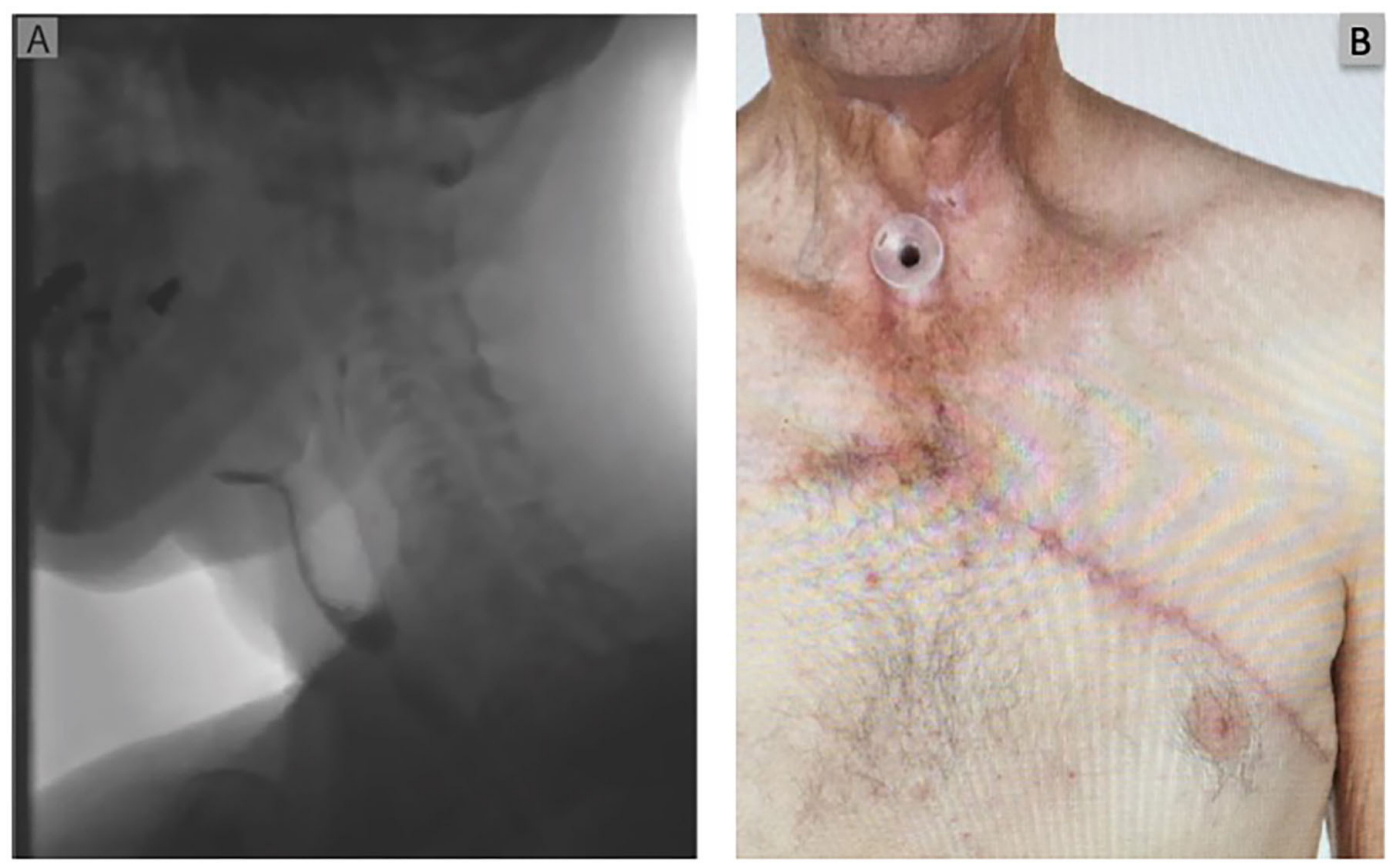
Post-operative Images and swallow at 6 weeks. **(A)** Depicts a post-operative swallow study demonstrating a patent pharyngeal tube with flow of contrast into the esophagus. **(B)** Post-operative clinical photograph demonstrating a well healed wound and donor site with no evidence of persisting pharyngocutaneous fistula.

The patient demonstrated a successful gastrografin swallow test at 6-weeks with no significant leak and wound fully healed. Six- months post-operatively, he continues to maintain adequate dietary intake independent of his percutaneous endoscopic gastrostomy (PEG) tube, a significant improvement noted by both the patient and ongoing speech pathology assessments (Figure 3).

## Discussion

The IMAP flap was first described by Yu et al. as a thin, pliable fasciocutaneous perforator flap based on the deltopectoral (DP) axis, for reconstructing tracheostomal and anterior neck defects (3). Unlike the DP flap which requires a wide based flap to ensure the capture of numerous perforators which subsequently restricts flap mobility, the IMAP flap can be islanded on a single perforator. This allows a wider arc of rotation and flap versatility, with the ability to achieve primary closure of the donor site and a single stage reconstruction. Meticulous dissection is required to free any adherent fascial bands around the IMA/V perforator, minimizing the risk of kinking as well as facilitating tension free mobilization of the islanded flap. Numerous anatomical studies have since improved the understanding of the utility and limitations of the IMAP flap (4–6). The flap can be raised on any of the first four IMA perforators (5), however the second has been shown to have the largest diameter perforator (1.6 ± 0.5 mm, range: 0.9–2.3 mm) and perfuse the largest skin dimensions (16 x 9 cm). The flap in this case was successfully raised on the left 2nd intercostal perforator. Although not required in this case, techniques can be employed to gain additional reach of the flap, such as removal of adjacent costal cartilages and distal ligation of the IMA with retrograde dissection (7, 8).

One of the main advantages of the IMAP flap is that in most patients, it is thin and pliable and therefore is less likely to obstruct around the stoma. Although the presented case demonstrates subfascial flap raising in a thin patient, studies have demonstrated the blood supply of the IMAP being mainly subdermal in nature and therefore suprafascial flaps have been reported for cases whereby the chest wall tissue is more bulky (9). In bulkier flaps, it may be possible to offset the tubularised skin paddle at 45°, to reduce the risk of the proximal portion of the flap obstructing the stoma. For buried flaps, like in our case, if direct closure isn't achievable, the skin from the de-epithelialized portion of the flap can be used as a graft. Consideration for the use in women or high BMI patients should be taken carefully, as not only is the flap potentially bulkier given the location, but closure of the donor site can significantly alter breast aesthetics.

Pre-operative imaging with CTA has been recommended to identify individual perforator location (2, 10). In the reported case, the CTA demonstrated bilateral intact IMA's, with only a large venous perforator in the right 2nd ICS, along with smaller arterial perforators in the right 1st and 3rd ICS. Intraoperative exploration demonstrated only small right sided arterial perforators in the 1st and 3rd ICS, insufficient for the required flap dimensions. Dissection proceeded to the left side, which demonstrated adequate caliber IMA/V perforator in the left second intercostal space in spite of the CTA findings. The most likely reason behind the discrepancy between the CT and clinical findings being that the imaging was performed in the venous phase. Although imaging is not essential, we would still advise either CTA or color duplex ultrasound, if available, which has been shown to ascertain perforator size and flow as part of the pre-operative planning (3, 11, 12).

This case demonstrates that even when raising larger IMAP flaps for circumferential defect reconstruction, tension free donor site closure is achievable with no donor site morbidity and an inconspicuous scar, achieving a functional and aesthetically acceptable outcome for such patients. In this particular case, the patient reported an improved quality of life with a healed wound and improved oral intake.

## Conclusion

The senior author has previously reported on the utility of the IMAP flap in reconstructing small to medium sized pharyngeal defects resulting from stricture release (2). This report demonstrates how this versatile, simple, and reliable flap can be used even for circumferential pharyngeal defects, with primary tension free closure of the donor site. Free tissue transfer is still our first-choice option for pharyngo-esophageal reconstruction, but following stricture release in a hostile, irradiated, and recipient vessel depleted neck, the IMAP flap is an excellent alternative option for reconstruction to be aware of, avoiding the known complications with irradiated vessels in head and neck reconstruction (13).

## Data Availability Statement

The original contributions presented in the study are included in the article/supplementary material, further inquiries can be directed to the corresponding author/s.

## Ethics Statement

Ethical review and approval was not required for the study on human participants in accordance with the local legislation and institutional requirements. The patients/participants provided their written informed consent to participate in this study. Written informed consent was obtained from the individual for the publication of any potentially identifiable images or data included in this article.

## Author Contributions

DG: development of surgical technique. NM and LS: contributions for manuscript text and figure preparation. All authors contributed to the article and approved the submitted version.

## Conflict of Interest

The authors declare that the research was conducted in the absence of any commercial or financial relationships that could be construed as a potential conflict of interest.
